# Wharton’s Jelly Bioscaffolds Improve Cardiac Repair with Bone Marrow Mononuclear Stem Cells in Rats

**DOI:** 10.3390/jfb16050175

**Published:** 2025-05-12

**Authors:** Luize Kremer Gamba, Laiza Kremer Gamba, Camila da Costa, Aline Luri Takejima, Rossana Baggio Simeoni, Isabella Cristina Mendes Rossa, Anna Clara Faidiga Silva, Julia Letícia de Bortolo, Marcos Antônio Denk, Seigo Nagashima, Carlos de Almeida Barbosa, Paulo Cesar Lock Silveira, Júlio César Francisco, Luiz César Guarita-Souza

**Affiliations:** 1 Experimental Laboratory of Institute of Biological and Health Sciences, Pontifícia Universidade Católica do Paraná (PUCPR), Curitiba 80215-901, PR, Brazilalinetakejima@yahoo.com.br (A.L.T.); rossanab@tecpar.br (R.B.S.); bella@gmail.com (I.C.M.R.); faidiga@gmail.com (A.C.F.S.); juliabortolo@gmail.com (J.L.d.B.); slidesdenk@gmail.com (M.A.D.); seigo.nagashima@pucpr.br (S.N.); cesar.souza@pucpr.br (L.C.G.-S.); 2 Graduate Program in Health Sciences, Centro Universitário para o Desenvolvimento do Alto Vale do Itajaí, Rio do Sul 89160-932, SC, Brazil; luizakremer@gmail.com; 3 Laboratory of Experimental Pathophysiology, Graduate Program in Health Sciences, Universidade do Extremo Sul Catarinense, Criciúma 88806-000, SC, Brazil; costa@gmail.com (C.d.C.); psilveira@unesc.net (P.C.L.S.); 4 Graduate Program in Pharmacy, Centro Universitário Curitiba—Unicuritiba, Curitiba 80220-181, PR, Brazil; carlos.b@pucpr.br

**Keywords:** Wharton’s Jelly, bioscaffolds, cardiac repair, bone marrow mononuclear stem cells, myocardial infarction, tissue engineering, regenerative medicine, rats

## Abstract

This study assessed the impact of implanting mononuclear stem cells and Wharton’s Jelly (WJ), either separately or together, on left ventricular dysfunction following myocardial infarction in Wistar rats. Functional and histopathological parameters were analyzed, and a rat model of left anterior descending coronary artery ligation was used. Treatments included an intramyocardial injection of 0.9% sodium chloride (control, *n* = 14), decellularized WJ (*n* = 12), bone marrow-derived mononuclear cells (BMMC) (*n* = 12), and bone marrow-derived mononuclear cells (BMMC) combined with WJ (*n* = 15). Echocardiography assessed the left ventricular function and ejection fraction over four weeks. Histological and immunohistochemical analyses with anti-factor VIII evaluated angiogenesis and collagen types I and III. The results showed no statistically significant effect on ventricular remodeling 30 days post-acute myocardial infarction (AMI). Moreover, the infarct area was significantly smaller in the BMMC + WJ group compared to the control group, suggesting a potential benefit in reducing myocardial scarring. BMMC + WJ therapy demonstrated potential for functional improvement and infarct size reduction 30 days post-infarction. Further studies are needed to confirm its therapeutic benefits.

## 1. Introduction

After acute myocardial infarction (AMI), there is a gradual process of loss of contractile myocytes involving the deterioration of ventricular function [1]. It is estimated that cardiovascular disease will be responsible for around 23.6 million annual deaths by 2030 worldwide [2].

Tissue engineering strategies using natural biomaterials have emerged as promising approaches for myocardial regeneration [3,4]. The goal is to develop scaffolds that reproduce the characteristics of a healthy myocardium [5]. Acellular extracellular matrix bioscaffolds derived from human Wharton’s Jelly (WJ) have shown potential in this field, promoting endogenous repair mechanisms such as angiogenesis and vasculogenesis while offering non-invasive collection methods with no ethical concerns [6].

Wharton’s Jelly is a connective tissue found in the human umbilical cord that contains numerous growth factors, significant amounts of various types of collagens (types I, III, IV, and V), hyaluronic acid, several proteoglycans, glycosaminoglycans, and proteins that play an important role in cell adhesion [7].

Recent research has shown that treatment with various stem cells including bone marrow-derived mononuclear cells (BMMC) could restore the cardiac function in preclinical models of myocardial infarction [7,8].

**Figure 1:**
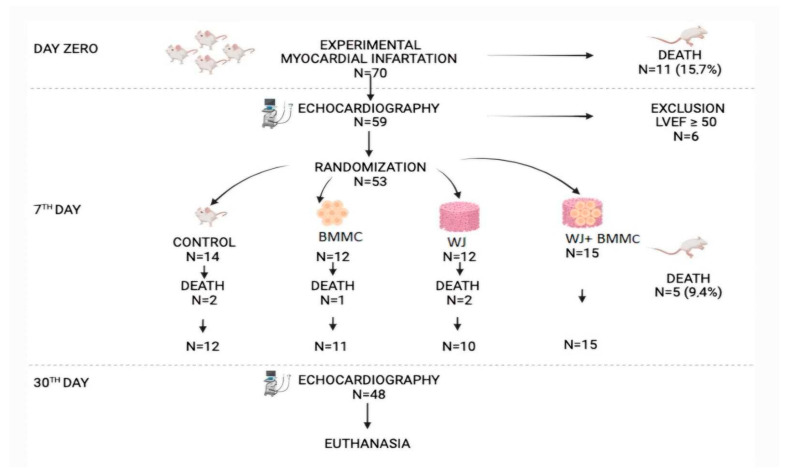
Schematic representation of this study. Myocardial infarction progression across the four experimental groups. Scaffold implantation procedure in the BMMC + WJ group.

A previous study demonstrated that a transepicardial injection of human BMMC enhanced cardiac function recovery and histomorphometric alterations in a rat model of AMI [9]. Therefore, the goal of this study is to evaluate the functional and pathological outcomes of bone marrow stem cell transplantation combined with acellular Wharton’s Jelly in a preclinical AMI model (Figure 1).

## 2. Materials and Methods

### 2.1. Animals and Treatment

Male Wistar rats weighing between 250 and 300 g were used, maintained under controlled temperature (22 ± 2 °C), humidity (50–60%), and a 12 h light–dark cycle. Animals had free access to water and standard chow. All experimental procedures were approved by the Institutional Animal Care and Use Committee of the Pontifical Catholic University of Paraná (PUCPR (02285/2. 70) and followed the guidelines of the Brazilian College of Animal Experimentation (COBEA).

The rats were randomly divided into 4 groups: control (*n* = 15), AMI with saline injection (control group); group (BMMC) (*n* = 15), AMI with bone marrow; group WJ (*n* = 15), AMI with Wharton’s Jelly; and group (BMMC + WJ (*n* = 15), AMI with bone marrow stem cells (BMMC) + Wharton’s Jelly.

And, the left coronary artery ligation (LAD) surgery was performed, as in our previous study [10]. Briefly, rats were anesthetized by ketamine (50 mg/kg, im) and xylazine (10 mg/kg, im). After opening the pericardium, a 7-0 silk suture was used to ligate the left coronary descending artery. The operations and all analyses were performed blinded. Rats were anesthetized for echocardiography detection on days 7 and 30 post-AMI, using the HD7 (Philips Medical Systems, Andover, MA, USA) with S12 sectorial (5–12 mHz) transducer. The animals were kept in cages and received commercial feed and water ad libitum.

### 2.2. Preparation of Acellular Wharton’s Jelly (WJ)

Acellular Wharton’s Jelly (WJ) was prepared according to Foltz et al. (2021) [11], using umbilical cords obtained with informed consent. The tissue was rinsed with phosphate-buffered saline (PBS) containing 2% penicillin/streptomycin, followed by the removal of blood vessels and excess connective tissue. The WJ was then fragmented and decellularized in a 0.01% sodium deoxycholate (SD) solution for 24 h at 37 °C under mechanical stirring. The processed WJ was preserved in PBS at 4 °C for further use.

### 2.3. Wharton’s Jelly (WJ) Harvesting Stem Cells and Isolation of Bone Marrow Stem Cells (BMMC)

The BMMC were obtained from the puncture of the autologous bone marrow of the rat iliac crest. The bone marrow (0.5 mL) was collected and separated by density gradient centrifugation (d = 1.077 g/m^3^—Ficoll-Hypaque-Sigma, St. Louis, MO, USA) using IMDI culture medium (Iscove’s modified Dulbecco medium) supplemented with antibiotics (1% penicillin and streptomycin), according to the technique described by Böyum. BMMC samples were resuspended and injected in multiple sites directly on the infarcted area and transition zone, as previously described by Takejima et al. [12]. Cell counts were determined using a Neubauer chamber and the cells were resuspended in sterile PBS at a concentration of 1 × 10^5^ cells/mL.

### 2.4. BMMC Transplantation and WJ Implantation

Seven days after acute myocardial infarction (AMI) and with an ejection fraction (EF) < 50%, the animals were randomly assigned to one of four groups: control, BMMC, WJ, and BMMC + WJ. Briefly, the animals were anesthetized again (50 mg/kg Ketamine and 10 mg/kg Xylazine), intubated, ventilated with a respirator, and supplemented with oxygen. A median transsternal sternotomy was performed. The BMMC group received 5 × 10^6^ mononuclear stem cells injected at multiple sites directly on the infarcted area and transition zone, as previously described. The BMMC + WJ group received a Wharton’s Jelly (WJ) implant, measuring approximately 2 cm × 3 cm, placed on the anterior surface of the left ventricle. The implant was sutured with 7-0 polypropylene over the ischemic area, identified under direct visualization. The control group received only saline solution through the transsternal sternotomy.

### 2.5. Tissue Microarray (TMA) and Immunohistochemistry (IHC) Flow Cytometry

For histological evaluation of infarcted heart tissue, hematoxylin–eosin (HE) staining was initially performed to assess myocardial fibrosis severity, followed by Masson’s trichrome staining. Tissue samples were prepared for Tissue Microarray (TMA) analysis, with selected areas marked for further investigation. Two 4 μm thick sections from the TMA blocks were placed onto electrically charged Star FrostTM slides and incubated overnight with primary antibodies, anti-Desmin (ab8592; (Abcam Inc, Toronto, ON, Canada), anti-CD68 (ab283654; Abcam), anti-CD31 (ab28364; Abcam), and anti-factor VIII (275376; Abcam), in a humidified chamber at 2–8 °C. Immunoreactivity was detected using DAB chromogen-substrate solution, followed by counterstaining with Harris hematoxylin. Positive and negative controls were included in each experiment. The slides were scanned using the Axio Scan.Z1 scanner (Carl Zeiss, Berlin, Germany), and approximately 25 random images were selected for analysis. Immunopositive areas were quantified using Image-Pro Plus software (version 4.5; Media Cybernetics, Rockville, MD, USA), with a “mask” function to standardize and automate object selection. The analysis was performed blindly, with the software randomly generating images to eliminate investigator bias. The immunopositivity data was then exported to Excel for further processing.

### 2.6. Flow Cytometry

Cells were washed and incubated with a blocking solution of PBS containing 1% normal rat serum for 10 min. Following this, cells were exposed to saturating concentrations of fluorochrome-conjugated antibodies for 30 min at 4 °C in the dark, then washed thoroughly. The antibodies used for analyzing human cell-surface markers included anti-CD34, anti-CD45, anti-CD105, anti-CD90, and anti-CD73, as specified in the Stem Kit (Beckman Coulter, Krefeld, Germany), according to the manufacturer’s recommended concentrations. Mouse IgGκ1 isotype controls (5 μg/mL, BD Pharmingen) were used to confirm the specificity of the staining. The samples were analyzed using a FACS Calibur system (BD Biosciences, San Jose, CA, USA), with data processing carried out using FACS CompTM software (v 4.1) (FlowJo LLC, BD, Franklin Lakes, NJ, USA). 

### 2.7. Scanning Electron Microscopy

The scaffold’s surface morphology was examined using scanning electron microscopy (SEM) (JEOL JSM-7900F, France). Before imaging, the specimens were coated with a thin gold layer using a sputter coater (Jeol, Tokyo, Sputter Coater Japan) and 10 kV of accelerated voltage and magnification of 50 to 200 was used to obtain the SEM images.

### 2.8. Statistical Analysis

Statistical analysis of data was performed using GraphPad Prism Version 8. Normality was verified by the Shapiro–Wilk test. “Normality was verified by the Shapiro–Wilk test. Normally distributed data were analyzed using one-way ANOVA with Tukey’s post hoc test, two-way ANOVA with Bonferroni post hoc test, or unpaired *t*-test, as appropriate. A significance level of *p* < 0.05 was considered statistically significant”.

## 3. Results

Seventy rats participated in this study, all of which underwent experimental infarction. Following this procedure, 11 animals died (15.71%). On the seventh day of the experiment, echocardiography was performed, and six animals had an ejection fraction >50%; therefore, they were excluded from this study and euthanized. Fifty-three animals were randomly divided using a paper draw, and the four study groups were subjected to biomaterial implantation or sodium chloride (control). Five animals did not survive the procedure (mortality rate of 9.43%), and 48 animals were followed without complications for 30 days until euthanasia at the end of the experiment. The groups were divided as follows: control group (*n* = 12), CT group (*n* = 11), WJ group (*n* = 10), and BMMC + WJ group (*n* = 15).

### 3.1. Tissue Characterization

After decellularization, key quality parameters were assessed, including the removal of cellular components and the preservation of the native extracellular matrix, both of which are critical for the proper characterization of a biological scaffold [13,14].

**Figure 2:**
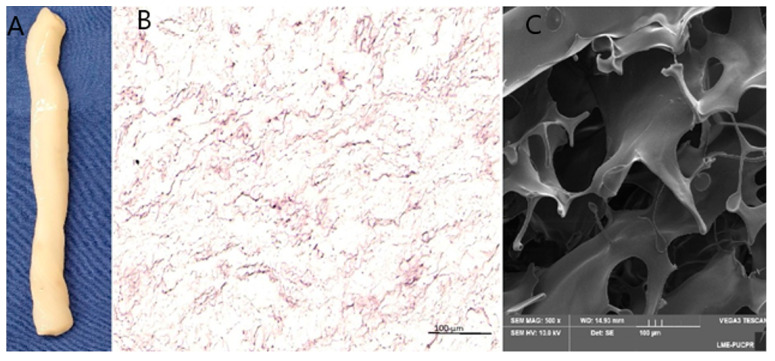
After decellularization, (**A**); Macroscopic observation of decellularized Wharton’s Jelly (WJ), which were evaluated with hematoxylin–eosin (HES) staining of paraffin-embedded sections (scale bar = 200 µm), (**B**); The structures were characterized with scanning electron microscopy (SEM) (scale bar = 100 µm), (**C**).

To evaluate the decellularization process, Wharton’s Jelly (WJ) was histologically analyzed using hematoxylin and eosin (H&E) staining and scanning electron microscopy (SEM) (Figure 2B,C). The results demonstrated the efficient removal of host cells and cellular fragments, with no visible nuclear residues. Additionally, the integrity of the mucous connective tissue structures and collagen fibers was preserved.

#### 3.1.1. Results of Cardiac Function Test—Inter-Group Evaluation

Combined Wharton’s Jelly and BMMC transplantation improved post-AMI cardiac function.

Regarding the analysis of LVEF function after seven days of AMI, no differences were identified between the groups; therefore, they were considered homogeneous (*p* = 0.434), as demonstrated in Table 1.

**Figure 3:**
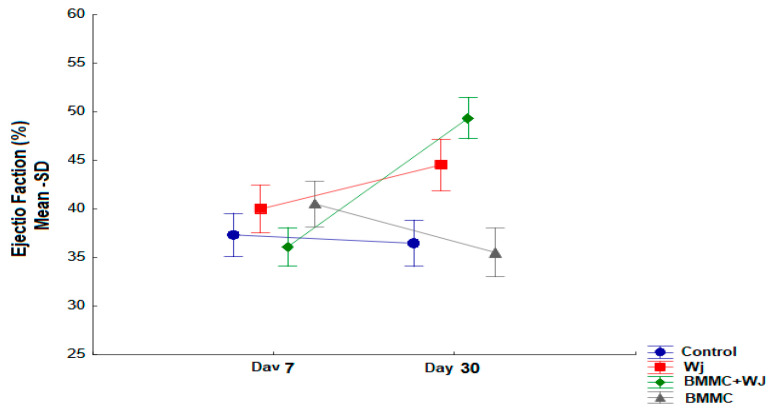
The 30th day of LVEF: LVEF after 30 days of coronary occlusion.

In the analysis at 30 days post-AMI, a statistical difference was identified between the groups (*p* < 0.001). When comparing the groups pairwise, it was observed that there was an increase in LVEF in the BMMC + WJ group compared to the control group (*p* = 0.001) and the CT group (*p* < 0.001), as demonstrated in Table 1 and Figure 3.

Intra-Group Assessment

In the intra-group analysis of the LVEF parameter, seven days after AMI, the values for the control group were 37.3% to 36.5% (*p* = 0.656). In the BMMC group, it was 40.5% to 35.5% (*p* = 0.071); in the WJ group, it was 40% to 44.5% (*p* = 0.051); and in the BMMC + WJ group, it was 36.1% to 49.4% (*p* = 0.001) (Table 1).

In the intra-group analysis of the pre-implant systolic volume, the control group exhibited values of 0.159 mL, whereas the BMMC group showed 0.182 mL, the WJ group alone demonstrated 0.097 mL, and the BMMC + WJ group displayed 0.198 mL. After 30 days, these values shifted, with the control group registering 0.194 mL, the BMMC group showing 0.142 mL, the WJ group at 0.177 mL, and the BMMC + WJ group recording 0.129 mL (Table 2).

**Figure 4:**
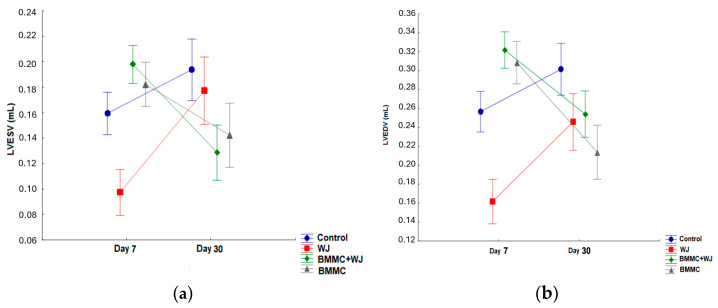
Comparison of groups two-by-two regarding LVESV—left ventricular end-systolic volume (**a**)—and LVEDV—left ventricular end-diastolic volume—7 (**b**) and 30 days after coronary occlusion.

In the comparison within each group, between day seven and thirty, only the BMMC + WJ group showed a statistical difference (*p* = 0.014) between the initial and VESV, —left ventricular end-systolic volume—and LVEDV—left ventricular end-diastolic volume (Figure 4).

##### Analysis of Collagen Levels and Infarct Area

To understand the progress of scaffolds + cell repair, we assessed the levels of collagen types I and III, which are predominant in healthy tissues, along with the percentage of each collagen type present in the scarred area.

A significant increase in collagen III levels was detected in the BMMC, WJ, and BMMC + WJ groups compared to the control group. Although a decrease in collagen I levels was observed in the BMMC + WJ group relative to the control, this difference did not reach statistical significance (*p* = 0.105) (Table 3).

**Figure 5:**
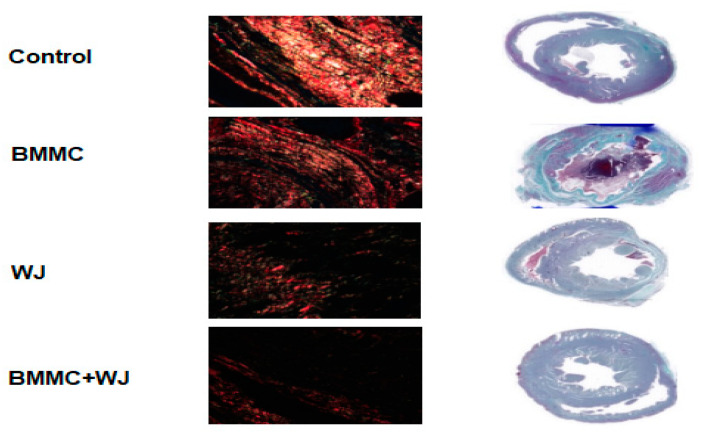
Infarct collagen content assessment after 30 days. Histopathological analysis using Picrosirius Red and Gomori’s trichrome staining performed thirty days after experimental infarction in the control group BMMC, WJ, and BMMC+ Wj. WJ: Wharton’s Jelly; BMMC: bone marrow mononuclear stem cells. Magnification 200×.

These results suggest a promising potential for tissue regeneration, supported by the facilitation of appropriate scar formation through the regulation of cardiac fibroblast functions, ultimately leading to enhanced cardiac function (Figure 5).

**Figure 6:**
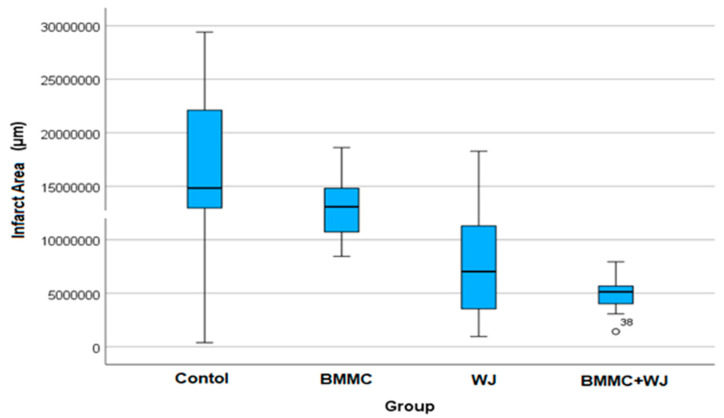
Infarct area content analysis after 30 days.

Histological analysis revealed that type I collagen was the most abundant in the scarred regions of the control group, while type III collagen was more prevalent in the BMMC group. However, these differences were not statistically significant. Cross-sections from the control, BMMC, WJ, and BMMC + WJ groups were stained with Masson’s trichrome to assess the infarcted area in each animal. After four weeks of infarction, there was no significant reduction in infarct size among the groups following 30 days of evaluation (Table 4 and Table 5, Figure 6).

**Figure 7:**
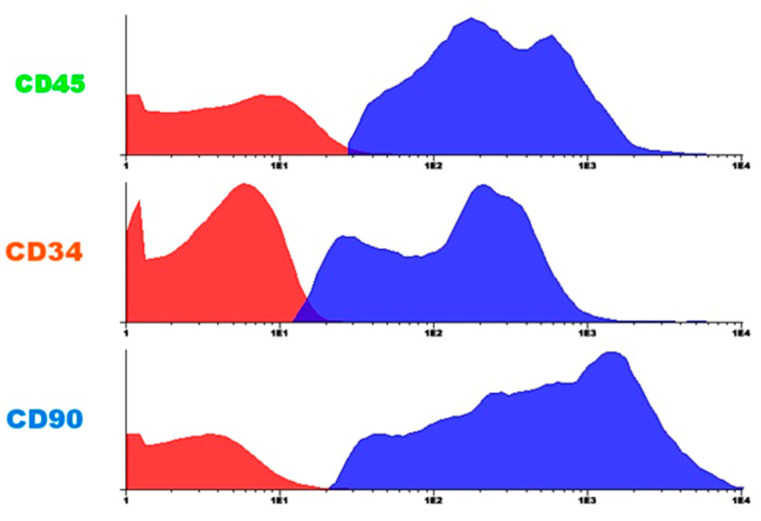
Characteristics of BMMC. Flow cytometry analysis of bone marrow-derived mononuclear cells (BMMC) was performed to assess the expression of specific markers. Cells were analyzed for hematopoietic markers (CD34, CD45) and mesenchymal markers (CD73, CD90, CD105). The findings revealed mesenchymal markers and expression of hematopoietic markers, confirming their mesenchymal stem cell profile.

A flow cytometric analysis of bone marrow-derived mononuclear cells (BMMC) was performed to validate their hematopoietic profile before implantation (Figure 7). 

##### Vascularization Evaluation

**Figure 8:**
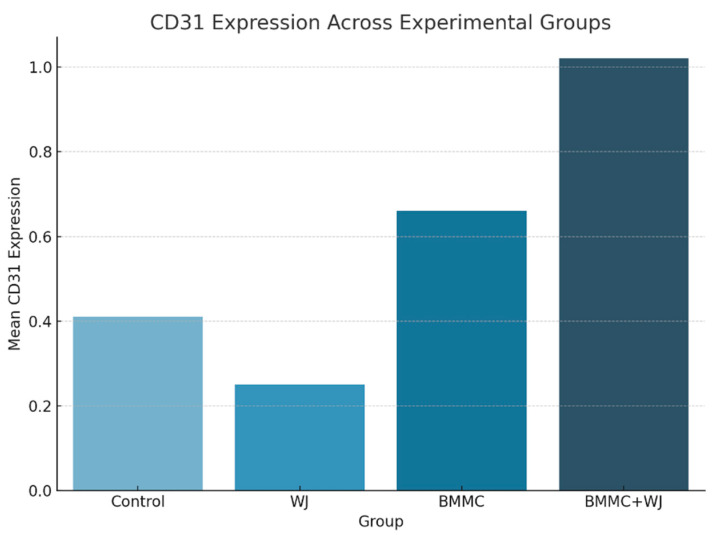
Mean CD31 expression levels across experimental groups.

To determine microvessel density, the specific marker for endothelial cells, CD31, was used. The results of the microvessel density analysis are summarized below. The microvessel density was highest in the BMMC + WJ group (1.02 ± 1.11) compared to the control group (0.41 ± 0.43), CT group (0.25 ± 0.23), and WJ group (0.66 ± 1.38) (Table 6, Figure 8). Although no statistical significance was found among the groups at 30 days post-treatment (*p* = 0.420), the trend indicates that the BMMC + WJ treatment may promote enhanced neovascularization compared to other groups (Figure 8). These findings are in accordance with previous studies suggesting that early increases in the microvessel density, as indicated by CD31 expression, are associated with improved neovascularization during the proliferative phase of tissue repair. Similar trends are reported in [15,16], highlighting that neovascularization is most pronounced during early healing stages and decreases as tissue maturation progresses. Further correlation analysis would be beneficial to investigate the interplay between proliferative activity and neovascularization in treated tissues, particularly to determine the role of the BMMC + WJ combination in sustaining vascular regeneration over time.

## 4. Discussion

Acute myocardial infarction (AMI) continues to be a leading cause of death and disability globally, highlighting the need for innovative treatments to promote cardiac repair. This study investigated the use of autologous bone marrow mononuclear cells (BMMC) in combination with decellularized Wharton’s Jelly (WJ) scaffolds as a regenerative strategy to enhance recovery following AMI.

Various strategies have been investigated to enhance cardiac regeneration, including the use of natural and synthetic scaffolds to support cell engraftment and tissue repair [17]. Previous studies have suggested that scaffolds provide a crucial microenvironment for cellular proliferation and structural support during tissue regeneration [18]. Notably, biomaterials mimicking the in vivo microenvironment have demonstrated significant potential in enhancing stem cell retention and engraftment [19,20].

The role of scaffolds in regenerative medicine has been exemplified by a study on cartilage regeneration in pigs, where acellular scaffolds with physiological anisotropy and bioactive extracellular matrices optimized host cell recruitment, adhesion, and proliferation, ultimately enhancing tissue regeneration [19]. Similarly, the myocardial microenvironment requires suitable scaffolding to support effective cardiac repair.

Stem cell-based therapy for AMI is primarily driven by paracrine signaling rather than direct differentiation into cardiomyocytes. Orlic et al. were the first to report that stem cells could differentiate into cardiomyocytes and significantly improve the cardiac function in a rodent infarction model [21]. However, subsequent studies, including our own, indicate that the isolated use of BMMC provides limited functional improvement, likely due to the inability to regenerate new cardiomyocytes in fibrotic areas [22].

Wharton’s Jelly, a naturally occurring extracellular matrix rich in growth factors, has been widely investigated for its regenerative capacity. Initially utilized in the treatment of chronic diabetic lesions, decellularized WJ retains various bioactive components that contribute to tissue repair [23,24]. Its unique composition makes it an attractive scaffold for cardiac tissue engineering, as it enhances cellular adhesion, proliferation, and paracrine factor secretion. Given that WJ is typically discarded after birth, its application in regenerative medicine presents an opportunity to repurpose a valuable biomaterial.

In our study, the decellularization of Wharton’s Jelly was confirmed by hematoxylin–eosin (HE) staining and scanning electron microscopy (SEM), demonstrating the complete removal of cells from the tissue. These results indicate that the preserved extracellular matrix of Wharton’s Jelly can serve as a suitable structural support for cell retention and tissue regeneration. The absence of cells in the decellularized material minimizes potential adverse immune responses and allows its safe application as a biological scaffold for cardiac tissue engineering.

In parallel with confirming the successful decellularization of Wharton’s Jelly, we also performed a flow cytometric analysis of bone marrow-derived mononuclear cells (BMMC) to validate their hematopoietic profile before implantation (Figure 7). This dual verification ensured both the scaffold, and the cellular component were appropriately characterized for cardiac regenerative therapy.

Wharton’s Jelly, an allogeneic biomaterial, is widely used in the fabrication of scaffolds for tissue engineering due to its ability to promote endothelialization. Furthermore, after the decellularization process, WJ exhibits no immunogenicity, making it a safe option for biomedical applications [25,26].

Our study showed that treatment with isolated BMMC did not lead to a statistically significant enhancement in the ejection fraction (EF), with the EF decreasing from an initial 40.5% to 35.5% at the end of this study (*p* = 0.071).

This finding aligns with previous research by Guarita-Souza et al., which reported EF stability 30 days post-intramyocardial stem cell application, with an initial EF variation from 21.79% to 18.60% (*p* = 0.4232) [20]. These results reinforce the hypothesis that BMMC alone may not be sufficient to induce cardiac regeneration.

However, the combination of BMMC with WJ scaffolds resulted in a significant increase in EF from 36.1% to 49.4% at 30 days post-AMI (*p* = 0.004), suggesting that WJ enhances cellular retention, angiogenesis, or paracrine signaling, thereby improving the cardiac function. Furthermore, the combined treatment led to a notable reduction in infarct size, as evidenced by the microscopy analysis of Gomori’s trichrome-stained heart slices. A significant decrease in collagen type I indices was also observed, which may contribute to enhanced cardiomyocyte survival post-ischemia.

The dynamic remodeling of extracellular matrix components plays a critical role in cardiac healing. Myofibroblasts produce large quantities of type III collagen in the early post-infarction phase (3–7 days), which is gradually replaced by type I collagen, forming a rigid scar. Our findings indicate that the presence of WJ scaffolds may modulate this process, thereby mitigating adverse post-infarction remodeling [27].

## 5. Conclusions

Our study suggests that combining autologous BMMC with decellularized WJ scaffolds has a synergistic effect in promoting cardiac functional recovery following AMI. This approach outperforms the use of BMMC alone by enhancing cellular retention, improving EF, and reducing the infarct size.

### Limitations

This study has some limitations. The follow-up period was limited to 30 days, which may not be sufficient to assess long-term cardiac remodeling. Additionally, the sample size was relatively small, and future studies with larger cohorts are necessary to confirm these findings. Further investigations should also explore additional biomarkers to elucidate the precise mechanisms by which WJ scaffolds contribute to myocardial repair.

## Figures and Tables

**Table 1:** Echocardiographic evaluations: representative flow parameters from each group before and 4 weeks after treatment.

*p* * (Compared Groups)		Standard Deviation	Mean	*n*	Group	Variable
		5.6	37.3	12	Control	LVEF (%) D7
		6.9	40.5	11	BMMC	
		8.1	40.0	10	WJ	
0.434		9.3	36.1	15	BMMC + WJ	
		5.7	36.5	12	Control	LVEF (%) D30
		8.2	35.5	11	BMMC	
		10.3	44.5	10	WJ	
<0.001	0.051	8.6	49.4	15	BMMC + WJ	

On the seventh day, echocardiographic assessment was performed prior to the implantation of the amniotic membrane and stem cells. On the thirtieth day, an echocardiographic evaluation was conducted following 30 days of coronary occlusion. LVEF refers to Left Ventricular Ejection Fraction; WJ stands for Wharton’s Jelly; and BMMC represents bone marrow mononuclear stem cells. * Student’s *t*-test for paired samples, the data are presented as mean ± standard deviation. *p*-values indicate statistically significant differences (*p* < 0.05).

**Table 2:** Assessing cardiac function using conventional echocardiography was performed across diverse groups in the present study.

Dimensional Parameters	Groups	7th	30th	*p* (7 × 30 th)
LVESV (mL)	Control (*n* = 12)	0.159 ± 0.046	0.194 ± 0.070	0.101
	BMMC (*n* = 11)	0.182 ± 0.063	0.142 ± 0.074	0.190
	WJ (*n* = 10)	0.097 ± 0.052	0.177 ± 0.177	0.079
	BMMC + WJ (*n* = 15)	0.198 ± 0.065	0.129 ± 0.129	0.014
LVEDV (mL)	Control (*n* = 12)	0.257 ± 0.065	0.301 ± 0.079	0.086
	BMMC (*n* = 11)	0.308 ± 0.091	0.214 ± 0.100	0.065
	WJ (*n* = 10)	0.162 ± 0.061	0.246 ± 0.102	0.006
	BMMC + WJ (*n* = 15)	0.322 ± 0.076	0.254 ± 0.098	0.079

Seventh day: echocardiographic evaluation before the amniotic membrane and stem cells’ implantation; Thirtieth day: echocardiographic evaluation after 30 days of coronary occlusion; LVESV: left ventricular end-systolic volume; LVEDV: left ventricular end-diastolic volume; WJ: Wharton’s Jelly; BMMC: bone marrow mononuclear stem cells. Data are shown as mean ± standard deviation. Test post-hoc de Bonferroni, Values of *p* < 0.05 denote statistical significance.

**Table 3:** Inter-group analysis of collagen types after 30 days post-infarction.

Variable	Group	*n*	Mean	SD	*p* *
Collagen I (%)	Control	11	95.4	6.8	20.7
	BMMC	11	84.6	16.6	5.49
	WJ	8	89.3	12.1	8.3
	BMMC + WJ	13	88.9	13.3	0.105
Collagen III (%)	Control	11	4.6	6.8	
	BMMC	11	15.4	16.6	
	WJ	8	10.7	12.1	
	BMMC + WJ	13	11.1	13.3	0.105

WJ: Wharton’s Jelly; BMMC: bone marrow mononuclear stem cells.; * Teste post-hoc de Bonferroni, Values of *p* < 0.05 denote statistical significance. The *p*-values for collagen types I and III are identical, as their combined percentage totals 100%.

**Table 4:** Inter-group analysis of infarct area (µm^2^) after 30 days post-infarction.

p* (Compared Groups)	SD	Mean	Group
<0.001	8,036,704.7	16,383,823	Control (*n* = 12)
	2,949,393.4	13,111,745	BMMC (*n* = 11)
	5,496,210.3	7,683,479.8	WJ (*n* = 10)
	1,806,522.8	4,938,556.6	BMMC + WJ (*n* = 15)

Values expressed in μm^2^. WJ: Wharton’s Jelly; BMMC: bone marrow mononuclear stem cells; * Values of *p* < 0.05 denote statistical significance.

**Table 5:** Two-by-two comparisons of the groups regarding the infarct area 30 days after infarct induction.

Groups Comparison	*p* *
BMMC + WJ × CONTROL	0.0004
BMMC + WJ × BMMC	0.0027
WJ × CONTROL	0.0448

Characterization of bone marrow-derived mononuclear cells (BMMC). WJ: Wharton’s Jelly. * Values of *p* < 0.05 denote statistical significance.

**Table 6:** Descriptive statistics for CD31 expression by group.

Group	*n*	Mean	SD	*p* *
Control	12	0.41	0.43	
WJ	11	0.25	0.23	
BMMC	10	0.66	1.38	
BMMC + WJ	15	1.02	1.11	*p* = 0.420

WJ: Wharton’s Jelly; BMMC: bone marrow mononuclear stem cells; * Values of *p* < 0.05 denote statistical significance.

## Data Availability

The original contributions presented in the study are included in the article, further inquiries can be directed to the corresponding author.
